# Proteomics of *Staphylococcus aureus* biofilm matrix in a rat model of orthopedic implant-associated infection

**DOI:** 10.1371/journal.pone.0187981

**Published:** 2017-11-09

**Authors:** Mei G. Lei, Ravi Kr. Gupta, Chia Y. Lee

**Affiliations:** Department of Microbiology and Immunology, University of Arkansas for Medical Sciences, Little Rock, Arkansas, United States of America; Purdue University, UNITED STATES

## Abstract

The matrix proteins of *Staphylococcus aureus* biofilm have not been well defined. Previous efforts to identify these proteins were performed using in vitro systems. Here we use a proteomic approach to identify biofilm matrix proteins directly from infected bone implants using a rat model of orthopedic implant-associated *S*. *aureus* infection. Despite heavy presence of host proteins, a total of 28 and 105 *S*. *aureus* proteins were identified during acute infection and chronic infection, respectively. Our results show that biofilm matrix contains mostly intracellular cytoplasmic proteins and, to a much less extent, extracellular and cell surface-associated proteins. Significantly, leukocidins were identified in the biofilm matrix during chronic infection, suggesting *S*. *aureus* is actively attacking the host immune system even though they are protected within the biofilm. The presence of two surface-associated proteins, Ebh and SasF, in the infected bone tissue during acute infection was confirmed by immunohistochemistry. In addition, a large number of host proteins were found differentially expressed in response to *S*. *aureus* biofilm formed on bone implants.

## Introduction

*Staphylococcus aureus* can cause a range of infections. Treatment for *S*. *aureus* infections is largely limited to conventional antibiotic therapy but failures are common due to emergence of antibiotic resistance strains as well as intrinsic antibiotic resistance attributed to biofilm formation. Biofilm is composed of multiple layers of bacteria encased and interconnected in an extracellular matrix that forms a complex three-dimensional structure, which provides protection against host defenses and limits the therapeutic efficacy of all currently available antibiotics [1, 2].

Biofilm formation is a complicated process that collectively includes three distinct phases—attachment, accumulation, and dispersal [2, 3]. Polysaccharide intracellular adhesion (PIA) is a major component in the biofilm matrix [4]. However, many *S*. *aureus* strains have been shown to form a PIA-independent biofilm composed of proteins and/or bacterial extracellular DNA (eDNA) [5–7]. These studies indicate that *S*. *aureus* biofilm matrix contains a large number of proteins but most of them have not yet been identified [6, 7]. However, these studies were all conducted by culturing bacterial cells under *in vitro* conditions and it is unclear whether the factors identified by *in vitro* studies reflect those present in biofilms formed *in vivo*. A recent study [8] revealed that only 26 of the more than 100 matrix proteins present in an *in vitro S*. *aureus* biofilm were recognized by antibodies from rabbits with experimental osteomyelitis, suggesting that matrix composition of biofilms formed *in vitro* is likely to be very different from biofilms formed *in vivo*. In this study, we address this issue by identifying matrix protein components in biofilms formed under *in vivo* conditions using a clinically relevant animal model of bone implant infection in rats. We identified a number of *S*. *aureus* proteins associated with biofilm matrix from bone implants during acute and chronic phases of infections. These proteins include secreted, surface proteins, but mostly intracellular proteins. Our results further indicate that direct identification of biofilm matrix proteins from infected tissues is feasible under our experimental condition.

## Results

### Development of a rat model of orthopedic-implant infection

To identify biofilm matrix proteins in vivo, we first developed a clinically relevant biofilm model to directly identify *S*. *aureus* biofilm matrix proteins in infected tissues. Recently, a post-arthroplasty *S*. *aureus* joint infection mouse model has been reported [9, 10]. We followed this model closely with two minor modifications. First, we used large rats based on the rationale that large rats would allow for larger implants to be placed in the femur than in mice, which would accommodate more bacteria to attach the implants thereby producing more biofilm materials. Second, we also employed stainless steel tubing in place of thin wire to increase biofilm volume by increasing the implant surface area and providing additional space within the implant. The groove at the distal end of the femur was exposed by skin incision and laterally displacing quadriceps-patellar complex. An opening was made at the center of the lower end of the femur and a piece of stainless metal tubing was inserted into the femoral canal. In pilot studies, we found tubing with 15G in size and 1-cm in length could be readily inserted into femoral canal. Tubing that is longer or larger in size was difficult to insert. In this model, we found presoaking the implants with 1x10^4^ CFU/ml for 30–60 min before placing into the femoral canal followed by inoculation of 50 CFUs of bacteria into the implant lumen would lead to visually marked swelling at day 6 post-infection with a bacterial load of 3x10^6^ to 2x10^8^ CFUs per implant. The same inoculum also led to low levels of bacterial load of 7x10^2^ to 2.3x10^4^ CFUs per implant at day 45. It should be noted here that presoaking implants in 1x10^9^ CFUs or inoculating higher inoculum (250 or 1,500 CFUs) into the implant lumen did not increase the bacterial load at day 6 or day 45 post-infection. The bacterial load and swelling at day 6 and day 45 post-infection were similar to the conditions defined as acute and chronic infection, respectively, in the post-arthroplasty *S*. *aureus* joint infection mouse model [9,10]. Thus, in this study, the time points at day 6 and 45 post-infection were used to study biofilm matrix proteins representing acute and chronic infection, respectively.

### Identification of biofilm matrix proteins from infected implants during an acute infection

To identify biofilm matrix proteins from an acute infection, we carried out the rat model described above. Two independent experiments were performed in which the implants were infected with *S*. *aureus* UAMS-1 or PBS. UAMS-1 was chosen because it is a clinical isolate from a patient with osteomyelitis [11]. All rats were able to walk on the surgical leg after day 1 post-infection. From day 2 to 6, only the control rats were able to walk normally on the surgical leg. All animals were sacrificed at day 6 post-infection at which time all infected animals had swollen joints while the control animals had no swelling. The implants were removed to harvest biofilm and determine bacterial load. In each experiment, the source of biofilm was pooled from implants of two infected rats with bacterial counts ranging from 7 x 10^6^ to 2.4 x10^7^ CFUs/implant. Protein fractions associated with the implants were collected for either exoprotein proteome (secretome) after sonication or SDS-extracted surface proteome (surfactome) analysis. It should be noted here that extraction by 4% SDS solution has been shown to release proteins that are associated with cell surface but not covalently anchored on the cell wall [12, 13]. The proteins were separated by SDS-PAGE and subjected to in-gel trypsin digestion followed by liquid chromatography-tandem MS (GeLC-MS/MS) analysis. Proteins were identified by Mascot search against UAMS-1 protein database [11, 14] using relatively stringent criteria of 99% protein threshold and a minimum of 2 peptides per protein sequence with 1% false detection rate. We found the vast majority of the proteins identified were rat proteins as expected and most bacterial proteins identified were low in abundance. We therefore considered positive identification of any protein that was present in both two independent experiments. We identified 11 and 17 UAMS-1-specific proteins from the secretome and surfactome, respectively (Tables 1 and 2), of which six proteins were found common to both proteomes.

**Table 1:** Proteins identified in secretome of acute phase infection.

ORF^a^ QV15_	gene	Identified Proteins	ID^b^	Mass (kDa)	Normalized count (expt 1/2)^c^		*P* value
					expt	control	
05465*	*otc*	ornithine carbamoyltransferase	KHF80801.1	38	7.6/28.6	0/0	0.114
13410*	*ldh2*	lactate dehydrogenase	KHF79648.1	34	21.8/5.3	0/0	0.121
05815	*ffh*	signal recognition particle	KHF80870.1	51	9.5/5.3	0/0	0.036
04425	--^d^	hypothetical protein [putative exported protein]	KHF80604.1	16	7.6/4.2	0/0	0.036
07005	*ebh*	matrix-binding protein	KHF81094.1	1135	5.7/4.2	0/0	0.010
13620*	*arcB*	ornithine carbamoyltransferase	KHF79689.1	38	1.9/7.4	0/0	0.117
02780	*adhA*	ethanol-active dehydrogenase/ acetaldehyde-active reductase	KHF80299.1	36	1.9/1.1	0/0	0.036
05335	*trxA*	thioredoxin	KHF80778.1	11	1.9/1.1	0/0	0.036
03870	*eno*	enolase	KHF80499.1	47	1.9/1.1	0/0	0.036
04315*	*glpQ*	glycerophosphodiester phosphodiesterase	KHF80583.1	35	0.99/2.1	0/0	0.060
08415	*uspA*	universal stress protein UspA	KHF81365.1	18	0.99/1.1	0/0	0.002

^a^ORF numbers are based on strain UAMS-1. ORF numbers with an asterisk (*) indicate proteins with a P value of >0.05. ORF numbers that are underlined indicate proteins also found in chronic secretome.

^b^The ID number is the Uniprot accession number.

^c^Two biological replicates were used to identify matrix proteins. The spectral counts were normalized based on total counts.

^d^—a gene name has not yet been assigned.

**Table 2:** Proteins identified in surfactome of acute phase infection.

ORF^a^ QV15_	gene	Identified Proteins	ID^b^	Mass (kDa)	Normalized count (expt 1/2)^c^		*P* value
					expt	control	
00825	*pflB*	formate acetyltransferase	KHF79941.1	85	120.0/118.0	0/0	<0.001
10795	*atpA*	ATP F0F1 synthase subunit alpha	KHF79165.1	55	26.7/20.9	0/0	0.007
02495	*tuf*	elongation factor Tu	KHF80244.1	43	20.0/17.7	0/0	0.002
00485	*adhE*	acetaldehyde dehydrogenase	KHF79877.1	95	14.7/11.2	0/0	0.009
08415	*uspA*	universal stress protein UspA	KHF81365.1	18	10.7/12.0	0/0	0.002
02780	*adhA*	ethanol-active dehydrogenase/ acetaldehyde-active reductase	KHF80299.1	36	8.0/13.6	0/0	0.031
05465*	*otc*	ornithine carbamoyltransferase	KHF80801.1	38	2.7/14.4	0/0	0.142
13410*	*ldh2*	lactate dehydrogenase	KHF79648.1	34	17.3/4.0	0/0	0.125
13610*	*arcC2*	carbamate kinase	KHF79687.1	34	1.3/7.2	0/0	0.142
05470*	*arcC1*	carbamate kinase	KHF80802.1	34	1.3/6.4	0/0	0.133
07020*	*tdcB*	threonine dehydratase	KHF81097.1	37	2.7/5.6	0/0	0.053
05335	*trxA*	thioredoxin	KHF80778.1	11	5.3/3.2	0/0	0.028
11465	*rpsE*	30S ribosomal protein S5	KHF79285.1	18	2.7/2.4	0/0	0.001
07025	*ald2*	alanine dehydrogenase	KHF81098.1	40	2.7/1.6	0/0	0.028
06820	*cspA*	cold-shock protein	KHF81061.1	7	4.0/3.2	0/0	0.006
03870	*eno*	enolase	KHF80499.1	47	1.3/1.6	0/0	0.004
05255*	*isdA*	heme transporter IsdA	KHF80763.1	39	2.7/0.8	0/0	0.102

^a^ORF numbers are based on strain UAMS-1. ORF numbers with an asterisk (*) indicate proteins with a P value of >0.05. ORF numbers that are underlined indicate proteins also found in chronic surfactome.

^b^The ID number is the Uniprot accession number.

^c^Two biological replicates were used to identify matrix proteins. The spectral counts were normalized based on total counts.

Only two surface or secreted proteins were identified in the secretome—Ebh, a very large extracellular matrix binding protein, which is surface associated and able to bind human fibronectin [15, 16], and a putative exported protein (QV15_04425). In the surfactome, IsdA, a surface anchored protein with LPXTG motif [17], was the only surface or secreted protein identified. In contrast, the majority of the proteins identified in both proteomes are cytoplasmic proteins or enzymes involved in cellular process and metabolism. Oct (ornithine carbomoyltransferase) and Idh2 (Lactate dehydrogenase) were the most abundant proteins in the secretome representing about half of all spectral counts, whereas PflB (formate acetyltransferase), which is involved in fermentation pathway, was the most abundant one representing almost half amount of the total spectral counts in the surfactome. Among these cytoplasmic proteins, TxrA (thioredoxin) and UspA (universal stress protein A), which were found in both proteomes, are potentially involved in virulence. TrxA is involved in redox homeostasis and removal of reactive oxygen species [18]. UspA has been shown to respond to stresses in other bacteria [19, 20] and to contribute to virulence in *Salmonella* [21]. The role of TxrA and UspA in *S*. *aureus* has not been studied. The presence of cytoplasmic proteins in the biofilm matrix is interesting but not surprising as these proteins have been reported to locate extracellularly in a variety of bacteria under different conditions including biofilms (reviewed in [22, 23]). A recent study also showed an increase in cytoplasmic proteins on the surface of *S*. *aureus* during biofilm formation in vitro [24].

### Identification of biofilm matrix proteins from infected implants during a chronic infection

To identify biofilm matrix proteins during a chronic infection, the infected rats were sacrificed at day 45. At the time of sacrifice, the *S*. *aureus* infected animals were slightly limping while walking. The joints were also less visually swelling compared to those of the acute phase. Because of low bacterial burdens, we pooled the proteins from 4 rats in each of the two experiments. The bacterial counts from individual implants were 7.0x102–1.6x10^4^ CFUs and 2.4x10^3^–2.3x10^4^ CFUs for the two independent experiments, respectively. We identified 33 and 72 proteins from the secretome and surfactome, respectively, in which 22 were in both fractions (Tables 3 and 4). Similar to the proteins identified during the acute phase infection, most of the proteins are cytoplasmic in nature. Furthermore, 8 of the 11 proteins found during acute infection were also found in chronic infection in the secretome (ORFs underlined in Tables 1 and 3), while 15 of the 17 proteins found in acute infection were found in chronic infection in the surfactome (ORFs underlined in Tables 2 and 4). The 6 proteins shared between secretome and surfacome in acute phase were also found in both proteomes in the chronic phase. Additional similarities to the acute phase profiles were that PflB was the predominant protein in surfactome and Oct was the predominant in secretome although Idh2 was no longer dominant in chronic infection. The repeated identification of the same proteins in different fractions and in both acute and chronic infection phases indirectly validates our experimental approach for identifying biofilm matrix proteins from infected tissues.

**Table 3:** Proteins identified in secretome of chronic phase infection.

ORF^a^ QV15_	gene	Identified Proteins	ID^b^	Mass (kDa)	Normalized count (expt 1/2)^c^		*P* value
					expt	control	
05465	*otc*	ornithine carbamoyltransferase	KHF80801.1	38	96.0/86.5	0/0	0.001
00825	*pflB*	formate acetyltransferase	KHF79941.1	85	21.35/15.6	0/0	0.011
02920	*mntA*	manganese ABC transporter substrate-binding protein	KHF80325.1	35	15.4/18.2	0/0	0.003
10785*	*atpD*	ATP F0F1 synthase subunit beta	KHF79163.1	51	23.7/11.2	0/0	0.054
01655	*ahpC*	alkyl hydroperoxide reductase	KHF80100.1	21	13.0/13.0	0/0	<0.001
10225	*lukA*	Leukocidin toxin subunit A	KHF79059.1	41	8.3/11.2	0/0	0.011
07765	*dnaK*	molecular chaperone DnaK	KHF81241.1	66	11.9/7.8	0/0	0.020
03870	*eno*	enolase	KHF80499.1	47	8.3/9.5	0/0	0.002
10220	*lukB*	Leukocidin toxin subunit B	KHF79058.1	39	7.1/8.6	0/0	0.005
13410	*ldh2*	lactate dehydrogenase	KHF79648.1	34	7.1/8.6	0/0	0.005
13685*	*sasF*	adhesin	KHF79702.1	70	2.4/9.5	0/0	0.119
06125*	*mutL*	DNA mismatch repair protein MutL	KHF80932.1	77	2.4/8.6	0/0	0.111
05255	*isdA*	heme transporter IsdA	KHF80763.1	39	4.7/6.1	0/0	0.007
02780	*adhA*	ethanol-active dehydrogenase/ acetaldehyde-active reductase	KHF80299.1	36	4.7/5.2	0/0	0.001
04845	*atl*	mannosyl-glycoprotein endo-beta-N-acetylglucosamidase (bifunctional autolysin)	KHF80684.1	138	4.7/5.2	0/0	0.001
07005	*ebh*	matrix-binding protein	KHF81094.1	1135	2.4/6.1	0/0	0.075
07020	*tdcB*	threonine dehydratase	KHF81097.1	37	3.6/5.2	0/0	0.017
12485*	*hlgA*	gamma-hemolysin subunit A	KHF79477.1	35	2.4/6.1	0/0	0.075
05335	*trxA*	thioredoxin	KHF80778.1	11	3.6/4.3	0/0	0.005
12495	*hlgB*	gamma-hemolysin subunit B	KHF79479.1	37	4.7/2.6	0/0	0.039
04315	*glpQ*	glycerophosphodiester phosphodiesterase	KHF80583.1	35	3.6/3.5	0/0	<0.001
11185	--^d^	iron citrate ABC transporter substrate-binding protein	KHF79234.1	37	3.6/3.5	0/0	<0.001
03850	*gap1*	glyceraldehyde-3-phosphate dehydrogenase	KHF80495.1	36	3.6/2.6	0/0	0.012
04330*	*pgi*	glucose-6-phosphate isomerase	KHF80586.1	50	3.6/0.9	0/0	0.121
02495	*tuf*	elongation factor Tu	KHF80244.1	43	2.4/1.7	0/0	0.012
11605*	--^d^	multidrug transporter	KHF79313.1	115	1.2/2.6	0/0	0.058
00485*	*adhE*	acetaldehyde dehydrogenase	KHF79877.1	95	1.2/2.6	0/0	0.058
08415*	*uspA*	universal stress protein UspA	KHF81365.1	18	1.2/2.6	0/0	0.058
08265*	*thrS*	threonyl-tRNA synthase	KHF81336.1	74	2.4/0.9	0/0	0.088
05080	*pdhD*	dihydrolipoamide dehydrogenase	KHF80729.1	49	1.2/0.9	0/0	0.012
13435	*fda*	fructose-1,6-bisphosphate aldolase	KHF79653.1	33	1.2/0.9	0/0	0.012
12010	*rpiA*	ribose 5-phosphate isomerase	KHF79390.1	26	1.2/0.9	0/0	0.012
05910*	*codY*	transcriptional repressor CodY	KHF80889.1	29	1.2/2.6	0/0	0.058

^a^ORF numbers are based on strain UAMS-1. ORF numbers with an asterisk (*) indicate proteins with a P value of >0.05. ORF numbers that are underlined indicate proteins also found in acute secretome.

^b^The ID number is the Uniprot accession number.

^c^Two biological replicates were used to identify matrix proteins. The spectral counts were normalized based on total counts.

^d^—a gene name has not yet been assigned.

**Table 4:** Proteins identified in surfactome of chronic phase infection.

ORF^a^ QV15_	gene	Identified Proteins	ID^b^	Mass (kDa)	Normalized count (expt 1/2)^c^		*P* value
					expt	control	
00825	*pflB*	formate acetyltransferase	KHF79941.1	85	497.5/469.9	0/0	<0.001
00485	*adhE*	acetaldehyde dehydrogenase	KHF79877.1	95	143.9/127.6	0/0	0.002
02495	*tuf*	elongation factor Tu	KHF80244.1	43	94.5/99.1	0/0	<0.001
07020	*tdcB*	threonine dehydratase	KHF81097.1	37	93.4/84.4	0/0	0.001
05465	*otc*	ornithine carbamoyltransferase	KHF80801.1	38	70.3/85.4	0/0	0.005
02780	*adhA*	ethanol-active dehydrogenase/ acetaldehyde-active reductase	KHF80299.1	36	80.2/67.9	0/0	0.003
07025	*ald2*	alanine dehydrogenase	KHF81098.1	40	63.7/67.0	0/0	<0.001
06145	*glpD*	glycerol-3-phosphate dehydrogenase	KHF80936.1	62	71.4/58.7	0/0	0.005
11040	*glmS*	glucosamine—fructose-6-phosphate aminotransferase	KHF79213.1	66	67.0/53.2	0/0	0.006
08265	*thrS*	threonyl-tRNA synthase	KHF81336.1	74	40.6/69.8	0/0	0.032
08415	*uspA*	universal stress protein UspA	KHF81365.1	18	63.7/44.1	0/0	0.016
05470	*arcC1*	carbamate kinase	KHF80802.1	34	59.3/44.1	0/0	0.011
13410	*ldh2*	lactate dehydrogenase	KHF79648.1	34	56.0/42.2	0/0	0.010
10955	*pyn*	thymidine phosphorylase	KHF79197.1	46	42.8/45.9	0/0	0.001
02490	*fusA*	elongation factor G	KHF80243.1	77	30.8/28.5	0/0	0.001
03870	*eno*	enolase	KHF80499.1	47	24.2/30.3	0/0	0.006
10785	*atpD*	ATP F0F1 synthase subunit beta	KHF79163.1	51	31.9/22.9	0/0	0.013
13435	*fda*	fructose-1,6-bisphosphate aldolase	KHF79653.1	33	25.3/23.9	0/0	<0.001
07765	*dnaK*	molecular chaperone DnaK	KHF81241.1	66	23.1/24.8	0/0	0.001
03850	*gap1*	glyceraldehyde-3-phosphate dehydrogenase	KHF80495.1	36	19.8/22.0	0/0	0.001
02920	*mntA*	manganese ABC transporter substrate-binding protein	KHF80325.1	35	16.5/22.9	0/0	0.013
08350	*pyk*	pyruvate kinase	KHF81352.1	63	19.8/18.4	0/0	0.001
05560	*ftsZ*	cell division protein FtsZ	KHF80819.1	41	16.5/17.4	0/0	<0.001
13625	*arcA*	arginine deiminase	KHF79690.1	47	13.2/19.3	0/0	0.017
10795	*atpA*	ATP F0F1 synthase subunit alpha	KHF79165.1	55	18.7/13.8	0/0	0.011
13440	*mqo2*	malate:quinone oxidoreductase	KHF79654.1	56	13.2/16.5	0/0	0.006
07205	*rpsA*	30S ribosomal protein S1	KHF81133.1	43	12.1/16.5	0/0	0.012
13610	*arcC2*	carbamate kinase	KHF79687.1	34	13.2/12.9	0/0	<0.001
01650	*ahpF*	NADH dehydrogenase (alkyl hydroperoxide reductase subunit F)	KHF80099.1	55	12.1/12.9	0/0	<0.001
04360	--^d^	hypothetical protein (putative fumarylacetoacetate hydrolase)	KHF80592.1	33	13.2/10.1	0/0	0.009
01655*	*ahpC*	alkyl hydroperoxide reductase	KHF80100.1	21	5.5/16.5	0/0	0.092
06140*	*glpK*	glycerol kinase	KHF80935.1	56	4.4/17.4	0/0	0.118
06520	*tkt*	transketolase	KHF81003.1	72	9.9/11.0	0/0	0.001
05170	*pyc*	pyruvate carboxylase	KHF80747.1	129	8.8/11.9	0/0	0.011
05910	*codY*	transcriptional repressor CodY	KHF80889.1	29	8.8/8.3	0/0	<0.001
00925	*ldh1*	lactate dehydrogenase	KHF79960.1	35	9.9/6.4	0/0	0.021
08465	*rpsD*	30S ribosomal protein S4	KHF81374.1	23	8.8/6.4	0/0	0.012
01055	--^d^	nitric oxide reductase	KHF81528.1	88	6.6/6.4	0/0	<0.001
08685	*pepV*	dipeptidase PepV	KHF81415.1	53	5.5/7.3	0/0	0.010
11185	--^d^	iron citrate ABC transporter substrate-binding protein	KHF79234.1	37	5.5/7.3	0/0	0.010
05070	*pdhB*	2-oxoisovalerate dehydrogenase	KHF80727.1	35	4.4/8.3	0/0	0.041
12355*	*narG*	nitrate reductase	KHF79452.1	140	2.2/9.2	0/0	0.122
01700	*guaB*	inosine-5-monophosphate dehydrogenase	KHF80108.1	53	4.4/6.4	0/0	0.017
11535	*rplB*	50S ribosomal protein L2	KHF79299.1	30	5.5/4.6	0/0	0.004
08830	*tal*	transaldolase	KHF81444.1	26	3.3/6.4	0/0	0.045
10575	*rsbW*	serine/threonine protein kinase	KHF79124.1	18	4.4/4.6	0/0	<0.001
00275	*sok*	myosin-cross-reactive antigen	KHF79836.1	68	4.4/4.6	0/0	<0.001
13145	*clpL*	Clp protease ClpL	KHF79599.1	78	3.3/5.5	0/0	0.029
05920	*rpsB*	30S ribosomal protein S2	KHF80891.1	29	3.3/5.5	0/0	0.029
10830	*upp*	uracil phosphoribosyltransferase	KHF79172.1	23	3.3/5.5	0/0	0.029
05080	*pdhD*	dihydrolipoamide dehydrogenase	KHF80729.1	49	5.5/2.8	0/0	0.047
00435	*deoC1*	deoxyribose-phosphate aldolase	KHF79867.1	24	4.4/3.7	0/0	0.004
10805	*atpF*	ATP F0F1 synthase subunit B	KHF79167.1	20	4.4/3.7	0/0	0.004
01080	--^d^	SAM-dependent methyltransferase	KHF79988.1	28	3.3/4.6	0/0	0.013
08420	*ackA*	acetate kinase	KHF81366.1	44	3.3/4.6	0/0	0.013
10225	*lukA*	Leukocidin toxin subunit	KHF79059.1	41	3.3/4.6	0/0	0.013
12370*	*nasD*	nitrite reductase	KHF79455.1	89	1.1/6.4	0/0	0.147
08595	--^d^	hypothetical protein	KHF81397.1	18	4.4/2.8	0/0	0.024
03380	*mgrA*	MarR family transcriptional regulator	KHF80406.1	17	3.3/3.7	0/0	0.001
04225*	--^d^	NADH dehydrogenase	KHF80565.1	44	2.2/4.6	0/0	0.053
09770	*ppaC*	inorganic pyrophosphatase	KHF78973.1	34	3.3/3.7	0/0	0.001
02355	*nupC*	nucleoside permease	KHF80217.1	44	2.2/4.6	0/0	0.053
02525	*ilvE*	branched-chain amino acid aminotransferase	KHF80250.1	40	3.3/2.8	0/0	0.004
12010	*rpiA*	ribose 5-phosphate isomerase	KHF79390.1	26	2.2/3.7	0/0	0.029
05335	*trxA*	thioredoxin	KHF80778.1	11	2.2/3.7	0/0	0.029
00310	*sirA*	iron ABC transporter substrate-binding protein	KHF79843.1	37	2.2/3.7	0/0	0.029
08390*	--^d^	universal stress protein	KHF81360.1	15	1.1/4.6	0/0	0.122
05990*	*infB*	translation initiation factor IF-2	KHF80905.1	78	1.1/4.6	0/0	0.122
09660*	*gatB*	glutamyl-tRNA amidotransferase	KHF78951.1	54	1.1/3.7	0/0	0.102
10585*	*rsbU*	serine/threonine protein phosphatase	KHF79126.1	38	1.1/2.8	0/0	0.073
11465*	*rpsE*	30S ribosomal protein S5	KHF79285.1	18	1.1/2.8	0/0	0.073
11510*	*rpmS*	50S ribosomal protein L29	KHF79294.1	8	1.1/2.8	0/0	0.073

^a^ORF numbers are based on strain UAMS-1. ORF numbers with an asterisk (*) indicate proteins with a P value of >0.05. ORF numbers that are underlined indicate proteins also found in acute surfactome.

^b^The ID number is the Uniprot accession number.

^c^Two biological replicates were used to identify matrix proteins. The spectral counts were normalized based on total counts.

^d^—a gene name has not yet been assigned.

Most significantly, bi-component leukocidins, LukAB and HlgAB, were found in the chronic secretome. The UAMS-1 genome encodes 5 leukocidin subunit genes, only the HlgC, which is a subunit of the leukocidin HlgBC, was not detected. However, the HlgC protein was detected by reducing the Mascot search stringency to 95% peptide threshold suggesting that it is likely present in the secretome as well. LukA subunit was also found in the SDS-extracted surfactome. Leukocidins are cytotoxins that target host immune cells [25]. The detection of the leukocidins in the biofilm matrix during chronic infection, but not during acute infection, suggests that these toxins may play a role against the host immune system at a later stage of implant bone infection. However, it is also likely that these toxins were also present during acute infection in a low quantity that were not detected by our methodology.

A few surface-associated or anchored proteins were found in the chronic infection. Ebh and IsdA, which were found in the acute phase of infection in the secretome and surfactome, respectively, were identified in the secretome during chronic infection. In addition, SasF, a cell wall-anchoring protein [26], and Atl, a bifunctional autolysin [27], were two other surface protein identified in the chronic secretome. In the chronic surfactome, we identified two surface proteins, Sok and SirA. Sok is a myosin cross reactive antigen that has been implicated in resistance to oxidative killing [28], whereas SirA is a receptor involved in the uptake of siderophore staphyloferrin B [29]. Except for Atl, none of these surface proteins have been previously shown to be involved in biofilm formation or maintenance. Atl has been shown to contribute to initial attachment phase of biofilm formation as well as by inducing cell lysis thereby promoting eDNA release [30, 31].

We noted several cytoplasmic proteins that are involved in virulence or stress response were identified (Tables 3 and 4). Four transcriptional regulatory proteins involved in virulence regulation were found in the chronic surfactome, which include CodY, MgrA, RsbW and RsbU, in which the latter two are involved in modulating the activity of the alternative sigma factor SigB. In addition to virulence regulation, these regulators have been shown to be responded to stress signals (reviewed in [32]). UspA and TrxA, as in the acute infection, were also found in both proteomes during chronic infection. In addition, AhpF and AhpC, which are subunits of alkylhydroperoxide reductase involved in detoxification of organic peroxides [33], were found in chronic surfactome. These proteins are potentially important for bacteria to survive in oxidative stress encountered during infection. Although the role of these cytoplasmic proteins in biofilm is unclear, their presence may indicate that they were upregulated, which may reflect bacterial adaptation during chronic infection.

### Ebh and SasF are present in biofilm matrix in an acute infection

**Fig 1:**
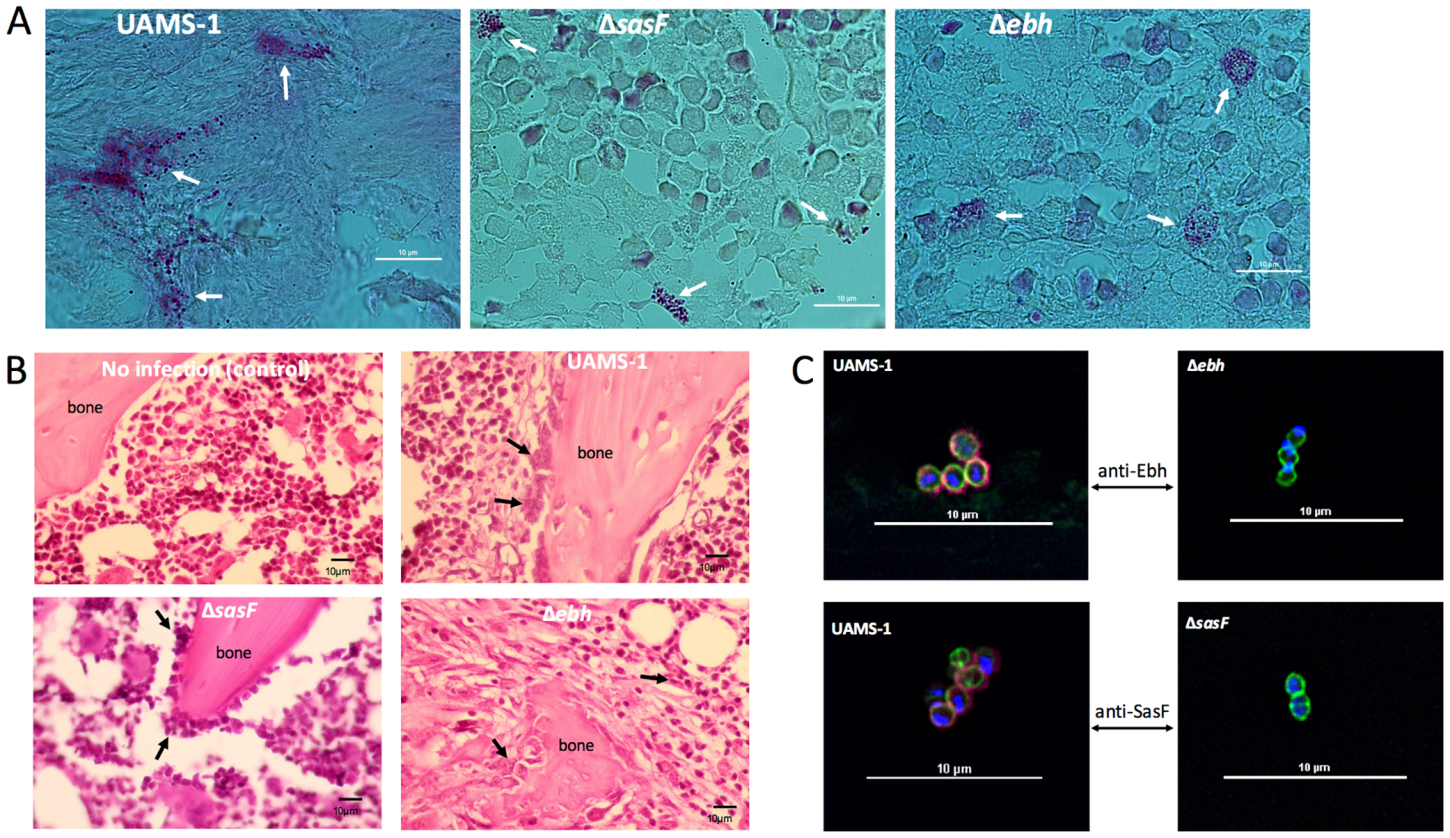
Demonstration of *S*. *aureus* in longitudinal bone sections of tissues surrounding implants infected with UAMS-1, *ebh* mutant or *sasF* mutant. Bars represent 10 μm in length. (A) Gram stain. Arrows indicate *S*. *aureus* cells. (B) Histopathology of infected tissues stained with H&E. Infiltration of inflammatory cells are indicated by arrows. (C) Immunohistochemistry of Ebh and SasF in biofilm matrix. Bone sections were stained with Hoechst dye (blue) for DNA, BODIPY-vancomycin (green) to reveal peptidoglycan, and anti-Ebh or anti-SasF along with secondary antibody conjugated to AlexaFluor-647 (red) to reveal Ebh or SasF. The images represent 2 to 6 fields of view for each mutant and control samples from duplicate experiments.

To validate that the proteins identified above are indeed present in the biofilm matrix during infection, we performed immunohistochemistry using antibodies against two surface proteins, Ebh and SasF. Ebh was identified in the secretome of both acute and chronic infections. In contrast, SasF was only identified in the chronic secretome. However, when peptide threshold was lowered to 95% we also found SasF in the acute secretome suggesting that SasF could also be present during acute infection. To detect these proteins, we used the rat post-arthroplasty joint infection model using short solid stainless wires essentially followed the mouse post-arthroplasty joint infection model as described by Bernthal et al [9]. Because the goal here was to demonstrate staphylococcal proteins on the surface of implants, we used small solid wires, instead of tubes, such that bacterial cells could be detected in bone thin section near the implant surfaces. At day 6 post infection, femurs with implant were harvested, fixed, decalcified and thin-sectioned longitudinally along the implant. The sections were subjected to hematoxylin and eosin (H&E) stain, Gram stain and immunofluorescent stain with anti-Ebh_N_ (N-terminal half) or anti-SasF. As shown in Fig 1A and 1B, staphylococci were easily identifiable in the bone tissue by Gram stain and the infected bone tissues were laden with inflammatory cells compared to the uninfected control. Both Ebh and SasF were expressed in the infected tissues whereas they were absent in the tissues infected with their isogenic mutant (Fig 1C). These results indicate that Ebh and SasF are expressed by staphylococci during an acute bone implant infection. However, the tissues infected with mutant strains had pathological changes similar to those infected with the wild type strain (Fig 1B). Since *ebh* and *sasF* deletion mutants were both capable of causing infection suggesting that lacking either one of them does not have apparent effect on bacterial survival in bone tissues. The detection of Ebh and SasF in the infected tissues suggests that Ebh and SasF are components of the biofilm matrix although our immunohistochemistry method did not provide high resolution to map whether they are surface-associated or secreted. The results also validate our proteomic approaches for identifying biofilm matrix proteins directly from infected host tissues. Furthermore, the results indicate that proteins detected at low spectral counts could not be simply regarded as false positives.

### Host proteins associated with *S*. *aureus* implants infection

We next identified host proteins that were induced or repressed by *S*. *aureus* infection. To this end, we compared host protein profiles with and without infection. We found 254 and 397 proteins had at least 2-fold difference with p-value of less than 0.05 in acute and chronic secretome profiles, respectively (S1 and S3 Tables). In the surfactome, 198 and 433 proteins were identified in acute and chronic samples, respectively (S2 and S4 Tables). The deferentially expressed genes were also analyzed by using DAVID software (S5 Table). Most of these proteins were induced by *S*. *aureus* infection. Proteins in various functional categories were identified but most belong to the category of immune response, cytoskeleton organization, cell differentiation, metabolism, signal transduction and transport. The large number of differentially expressed host proteins identified in this study suggests that *S*. *aureus* infection results in a profound change in host protein production. This is not surprising as the host is expected to mount a strong defense response in an effort to clear *S*. *aureus* but the bacterial persist in biofilm. While some of these proteins are not known to play a role in bacterial infection, some are known. For example, peptidoglycan recognition protein 1, which has amidase activity and binds to peptidoglycan in response to bacterial infection [34, 35], was highly induced (at least 3.8-fold) in all four profiles (although the p value in acute secretome is 0.064). Lipopolysaccharide binding protein, (LBP), which have been shown to interact with lipoteichoic acid of *S*. *aureus* [36], were also detected in both chronic samples. LBP has also been shown to be induced by *S*. *aureus* in an experimental intramammary infection during clinical mastitis [37]. As bacterial cell wall components are important immunogens recognized by host receptors, these results further indirectly validate our results. While the majority of the proteins were induced by *S*. *aureus* infection, we found some proteins were suppressed. Of these, the most noticeable were collagen components and related proteins involved in collagen formation, particularly in chronic infection samples. Concomitantly, collagen degradation enzymes were found to be significantly increased. Taken together, these results suggest that *S*. *aureus* infection in bone results in reduction in collagen, a main component in bone, by inducing host enzymes that degrade collagen. However, it is possible that production of staphylococcal proteases could also play a role.

## Discussion

The composition of matrix protein of biofilms formed during an *S*. *aureus* infection has not been reported. Limited biofilm materials that could be recovered from infected tissues, heavy contamination with host proteins, and availability of sensitive proteomic analytical methods are likely the limiting factors for assessing biofilm matrix composition directly from biofilms obtained from an in vivo infection. In this study, we attempted to identify proteins from biofilms formed on bone implants during acute and chronic phases of infection in rats by using GeLC-MS/MS. To obtain sufficient biofilm materials for analyses, we used large rats to accommodate implants that are too large for mice. By using this strategy, we were able to obtain enough biofilm materials for bacterial protein identification from implants harvested during acute infection as well as chronic infection. Despite the vast majority of the proteins identified were rat proteins, we were able to identify many bacterial proteins. In the negative control experiments without an infection, we identified only rat proteins without *S*. *aureus* proteins. Thus, we are confident that most, if not all, bacterial proteins that we identified in this study were of *S*. *aureus* and not false positives due to heavy contamination of rat proteins. Furthermore, we have used immunofluorescent staining against Ebh and SasF to verify that *S*. *aureus* proteins were indeed expressed in the rat bone tissue surrounding the infected implants. However, because of the heavy presence of host proteins, it is very likely that *S*. *aureus* proteins presence in a small amount were not identified. This limitation is difficult to avoid with the current technology. Thus, our results are likely to underestimate the number of staphylococcal proteins present in the matrix. This could also explain that SasF was positively identified in the immunofluorescent stain but was only identified in the acute infection when less stringent Mascot search criteria were used.

In *S*. *aureus*, polysaccharides, eDNA and proteins have been found in biofilm matrix under in vitro conditions but the components vary depending on the environment that the organism resides [38]. In this work, we used sonication and SDS to release proteins from the matrix of staphylococcal biofilm formed on metal implants. Sonication has been shown to be an effective method of removing biomatrix materials from biofilm [39], whereas SDS is an effective agent to extract surface-associated proteins without lysing staphylococci [12, 13]. We therefore assume that sonication should be able to release most proteins secreted/released into the extracellular biofilm matrix and the subsequent SDS treatment should release those that adhere or non-covalently associate with the bacterial surfaces. We found more proteins in the SDS fraction than the sonication fraction suggesting that many proteins are secreted/released but associated with the cell surface. We also found that the number of bacterial proteins identified in the chronic phase was much more than that in the acute phase suggesting that *S*. *aureus* continues to secrete diverse proteins once it establishes a long-termed chronic infection. This transition in protein profile suggests that *S*. *aureus* is actively modulating its environment during the course of the infection. Most significantly, leukocidins (LukAB, HlgAB and likely HlgCB) were identified in the biofilm matrix during chronic infection although UAMS-1 is capable of producing many other extracellular toxins and virulence factors with a notable exception of alpha-toxin due to a nonsense mutation [40]. Leukotoxins have been shown to target neutrophils, macrophages and monocytes [25]. In addition, HlgAB has also been shown to lyse erythrocytes, which are abundant in bone marrow, to release hemoglobin for iron source [41]. The presence of leukocidins in the biofilm matrix suggests that they play a pivotal role in bacterial survival and persistence in biofilm during bone infection by damaging host immune cells. Lysis of host immune cells would also trigger inflammatory response that could further cause tissue damage [25]. Our results further indicate that *S*. *aureus* not only protects itself by physically shielding from host immune system within the biofilm matrix but also actively releasing toxins to attack the host defense system. This is consistent with a recent study showing that LukAB as well as alpha-toxin actively produced from *S*. *aureus* USA300 LAC biofilms cause macrophage dysfunction in a murine model of orthopedic implant biofilm infection [42]. Leukotoxins and alpha-toxin have also been detected in biofilm using a human epidermal model [43].

The majority of the bacterial proteins we identified are cytoplasmic proteins. Cytoplasmic proteins have been found to be secreted extracellularly in many bacteria, including *S*. *aureus* [23, 44–46]. Our results are consistent with studies showing that many cytoplasmic proteins are found in the matrix of *S*. *aureus* biofilm formed in vitro [8, 24, 47]. In particular, Foulston et al. [24] show that most proteins found in the matrix from in vitro static *S*. *aureus* biofilms are cytoplasmic proteins. Thus, cytoplasmic proteins are likely the major matrix components of in vitro as well as in vivo biofilms. Bacterial programmed cell death during biofilm formation has been shown to release DNA that contributes to biofilm matrix formation [5]. This regulated autolysis is likely to result in release of intracellular proteins. However, studies have shown that cell lysis cannot explain the extracellular accumulation of cytoplasmic proteins but the molecular mechanisms involving in such secretion are still unknown [48, 49]. It has been reported that some of the released cytoplasmic proteins function as adhesins that interact with host cells [23]. However, as we found that many of these cytoplasmic proteins were only found in mature biofilm matrix during chronic infection, it is unlikely that they serve as adhesins for initial biofilm formation. Thus, other than moonlighting as components of biofilm matrix, whether these cytoplasmic proteins play additional roles awaits further investigation.

We found a total of 5 surface proteins (Ebh, SasF, Atl, IsdA and Sok) in this study of which Ebh and SasF were shown to be in the biofilm matrix using immunohistochemistry. However, both *ebh* and *sasF* mutants showed similar pathological changes as the wild type suggesting that they are not essential for implant biofilm formation although we did not measure the bacterial load on the implants. More than a dozen of *S*. *aureus* surface proteins have been demonstrated to be involved in biofilm formation in vitro [6, 7]. Strain UAMS-1 possesses eight of these proteins in the genome, which include FnbA, ClfA, ClfB, SdrC, SasC, Spa, Atl and SraP [14] but only Atl was identified in our study. Whether the other four surface proteins we identified play a role in biofilm formation/maintenance remained to be studied.

Although the main goal of this study is to identify bacterial proteins during infection, the proteomic approach also allowed us to identify host proteins that are affected by *S*. *aureus* infection. By analyzing host proteins differentially expressed globally, we provided a snapshot of host proteomic changes upon bone implant infection at both acute and chronic phases. We found profound changes in host proteins upon infection. This is not surprising because host is expected to mount a strong immune defense system to eliminate the invader in response to bacterial infection. However, although many proteins related to immune responses are to be expected, we found many more proteins that have not been implicated in response to *S*. *aureus* infections. It should be noted that our method in which sonication and 4% SDS were used to release bacterial proteins may also cause lysis of host cells. Thus, the host proteins shown in S1 to S4 Tables may also contain released proteins due to cell lysis. Nevertheless, our results provide a useful information for further in-depth studies on host-bacteria interaction during biofilm-related *S*. *aureus* infection.

## Materials and methods

### Ethics statement

All animal experiments were approved by the Institutional Animal Care and Use committee (IACUC) at the University of Arkansas for Medical Sciences.

### Bacteria

*S*. *aureus* UAMS-1 [11] and its derivatives were grown routinely in tryptic soy medium. *E*. *coli* XL-Blue, used for cloning purpose, was grown in Luria-Bertani medium. Antibiotics were added to the culture medium when necessary, at final concentrations of 10 μg/ml for erythromycin and chloramphenicol and 100 μg/ml for ampicillin. The primers used in this study are listed in S6 Table. To construct UAMS-1 Δ*ebh*::*ermC* mutant (CYLA25) and Δ*sasF*::*ermC* mutant (CYLA23), deletions were made by overlapping PCR using primer sets ebh1-4 and sasF1-4 (S6 Table), respectively. Each of the resulting fragment contains a NotI sequence in place of the deleted sequence. The amplified fragments were verified by sequencing and cloned into pJB38 to which a NotI fragment containing the *ermC* gene amplified by primer pairs erm11 and erm12 (S6 Table) from pCN44 [50] and cloned in pGEMT-easy (Promega, Madison WI) was inserted. Mutants were obtained by allele replacement as described previously [51]. The mutations were confirmed by PCR.

### Rats and infection model

#### Rat orthopedic implant-associated infection model

Sprague-Dawley (Harlan Inc., Indianapolis, IN) male adult rats of 420–520 g were anesthetized via inhalation of isoflurane (2%). Buprenex (15 μg) was injected subcutaneously and dispersed by gentle rubbing. A skin incision was made over the right knee to access the distal femur through a medical parapatellar arthrotomy with lateral displacement of the quadriceps-patellar complex. The femoral intramedullary canal was reamed with an 18-gauge (18G) needle and further opened with a hand-held drill (size 13G drill bit). One piece of 1-cm long orthopaedic-grade hypodermic stainless steel tubing (size 15G; Vita Needle Co., Needham, MA) that had been soaked with 1x10^4^ CFU/ml of *S*. *aureus* UAMS-1 or with PBS (control) was surgically placed into the femur. A total of 50 CFUs in 5 μl PBS or PBS alone was then inoculated in the lumen of the implant before the quadriceps-patellar complex was reduced to midline and the surgical site closed by sutures. Rats were sacrificed at 6 or 45 days after infection, representing acute and chronic infection, respectively. Implants were removed to collect biofilm and bacteria for matrix proteins and cell surface-associated proteins isolation.

#### Rat post-arthroplasty joint infection model

Sprague-Dawley male rats weighing 250–300 g were used in this model essentially as described by Bernthal et al. [9] in their mouse post-arthroplasty joint infection model. A 2-cm hypodermic stainless steel rod (size 23G; Vita Needle Co.) presoaked with 1x10^4^ CFU/ml *S*. *aureus* UAMS-1, Δ*ebh* or Δ*sasF* mutant was inserted into the medullary cavity with 1 mm protruding out. The protruding ends were infected with 2 μl of bacterial suspension (20 CFUs) in PBS before the quadriceps-patella complex was returned to midline and the surgery site closed with suture.

### Protein isolation and identification

The implants were removed, rinsed in 1 ml sterile saline and transferred to a 5ml-culture tube containing 1 ml sterile saline with protease inhibitor cocktail (Roche Diagnostics, Indianapolis, IN). The materials in the tubing were then dislodged with a wire and subjected to sonication in a Misonix Sonicator 3000 (Thermo Fisher Scientific, Waltham, MA) using micro-tip with four 10-s pulse at full power and 30-s intervals in between on ice. The mixtures were centrifuged at 6,000xg. The supernatants, representing exoprotein proteome fraction (secretome), were TCA precipitated and respuspended in SDS sample buffer. The pellets were resuspended in 4% SDS (10 μl per implant), incubated at room temperature for 1 h, centrifuged at 18,000xg, and the supernatants were defined as the surface proteome fraction (surfactome). The proteins in each fraction were analyzed in 4–15% gradient SDS-PAGE (Bio-Rad) and stained with Coomassie Blue G250, the gel lanes were equally cut into 3 mm slices and subjected to in-gel trypsin digestion as follows. Gel slices were destained in 50% methanol (Thermo Fisher), 100 mM ammonium bicarbonate (Sigma, St Louis, MO), followed by reduction in 10 mM Tris(2-carboxyethyl)phosphine (Pierce, Rockford, IL) and alkylation in 50 mM iodoacetamide (Sigma). Gel slices were then dehydrated in acetonitrile (Thermo Fisher), followed by addition of 100 ng porcine sequencing grade modified trypsin (Promega) in 100 mM ammonium bicarbonate (Sigma) and incubation at 37°C for 12–16 hours. Peptide products were then acidified in 0.1% formic acid (Pierce). Tryptic peptides were separated by reverse phase Jupiter Proteo resin (Phenomenex, Torrance, CA) on a 200 x 0.075 mm column using a nanoAcquity UPLC system (Waters, Milford, MA). Peptides were eluted using a 30 min gradient from 97:3 to 65:35 buffer A:B ratio (Buffer A = 0.1% formic acid, 0.5% acetonitrile; buffer B = 0.1% formic acid, 99.9% acetonitrile). Eluted peptides were ionized by electrospray (2.15 kV) followed by MS/MS analysis using higher-energy collisional dissociation (HCD) on an Orbitrap Fusion Tribrid mass spectrometer (Thermo Fisher) in top-speed data-dependent mode. MS data were acquired using the FTMS analyzer in profile mode at a resolution of 240,000 over a range of 375 to 1500 m/z. Following HCD activation, MS/MS data were acquired using the ion trap analyzer in centroid mode and normal mass range with precursor mass-dependent normalized collision energy between 28.0 and 31.0. Proteins were identified by database search using Mascot (Matrix Science, London, UK) with a parent ion tolerance of 3 ppm and a fragment ion tolerance of 0.5 Da. Scaffold (Proteome Software, Portland, OR) was used to verify MS/MS based peptide and protein identifications. Protein probabilities were assigned by the Protein Prophet algorithm [52]. Functional enrichment analysis of the host proteins was carried out using DAVID (https://david.ncifcrf.gov/) software program [53].

### Histology and immunohistochemistry analysis

At day 6 post-infection, whole femurs with implant were harvested, fixed in 10% formalin for 3 d, rinsed 3 times with Milli-Q water, and decalcified in Super Decalcifier I solution (Polysciences, Warrington, PA). The decalcified femurs were sent to Nationwide Histology (Veradale, WA) for paraffin embedding, mounting, thin-sectioning (4 μm thick, longitudinal to the implant), and routine hematoxylin-eosin (H&E) and Gram staining. To detect Ebh and SasF in infected tissues, the procedure for immunohistochemistry analysis was essentially as described by Cheng et al. [16]. Briefly, sections on microscope slides were treated with xylene (5 min, 3 times), followed by 100% ethanol (10 min, 2 times) then by 95, 70 and 50% ethanol for 5 min each. After rinsing with deionized water, the samples were rehydrated with PBS for 10 min and immersed in Uni-Trieve solution (Innovex Biosciences, Richmond, CA) for antigen retrieval for 30 min. The slides were blocked in human IgG (Sigma), followed by primary specific rabbit antibody (1:5000) of anti-Ebh [16] or anti-SasF [26] and AlexaFluor-647 conjugated goat-anti-rabbit secondary antibody (1:10,000; Invitrogen, Waltham, MA) for 1 h each in 3% BSA, 0.1% Tween 80 in 1x PBS and rinsed 3 times with 1x PBS in between. To reduce the background, primary antibodies were pre-absorbed with respective mutant strains. Finally, the slides were placed in 1x PBS containing 10 μg/ml Hoechst dye (Invitrogen) and 1 μg/ml BODIPY-vancomycin (Invitrogen) for 5 min. The slides were washed with PBS and mounted in N-propylgallate (Sigma) and viewed under a laser confocal microscope (Nikon Eclipse Ti, Nikon Instruments Inc., Melville, NY).

## Supporting information

S1 Table Host proteins by sonication affected by *S*. *aureus* during acute phase of infection. (XLS)

S2 Table Host proteins by SDS-extraction affected by *S*. *aureus* during acute phase of infection. (XLS)

S3 Table Host proteins by sonication affected by *S*. *aureus* during chronic phase of infection. (XLS)

S4 Table Host proteins by SDS-extraction affected by *S*. *aureus* during chronic phase of infection. (XLS)

S5 Table Functional enrichment analysis of host proteins affected by *S*. *aureus* during infection. (DOCX)

S6 Table Oligonucleotide primers used in this study. (DOCX)
