# Supplementary material for: Proteomics of Staphylococcus aureus biofilm matrix in a rat model of orthopedic implant-associated infection
Source: PLoS One. 2017 Nov 9;12(11):e0187981. doi: 10.1371/journal.pone.0187981 (PMC5679556; doi:10.1371/journal.pone.0187981)
Supplement: S5 Table — (DOCX) [file pone.0187981.s005.docx]

Table S5. Functional enrichment analysis of host proteins affected by *S. aureus* during infection

(A): Host proteins by sonication affected by *S. aureus* during acute phase of infection. A total 9 gene functional groups were identified by the Gene Functional Classification tool using DAVID software. The associated biology terms with each group are manually summarized based on gene-term enrichment buttons provided for each functional group.

Gene functional group no. Associated biology Group Enrichment Score

1 RAS small GTPase 21.8

2 Amine metabolism 17.98

3 cell replication 17.7

4 Microtubules 16.8

5 calcium binding protein 15.4

6 Cytokine/cell locomotion 15.0

7 Molecular chaperone 14.4

8 Macromolecular Catalytic activities 11.3

9 Cell transport 6.8

(B): Host proteins by SDS-extraction affected by *S. aureus* during acute phase of infection. A total 5 gene functional groups were identified by the Gene Functional Classification Tool using DAVID software. The associated biology terms with each group are manually summarized based on gene-term enrichment buttons provided for each functional group.

Gene functional group no. Associated biology Group Enrichment Score

1 Calcium binding protein 6.6

2 RAS small GTPase 5.8

3 Protein synthesis 5.7

4 Cell signaling/phosphorylation 4.7

5 Cell vesicular transport 3.8

(C): Host proteins by SDS-extraction affected by *S. aureus* during chronic phase of infection. A total 13 gene functional groups were identified by the Gene Functional Classification tool using DAVID software. The associated biology terms with each group are manually summarized based on gene-term enrichment buttons provided for each functional group.

Gene functional group no. Associated biology Group Enrichment Score

1 Metal binding proteins 24.1

2 Kinase activity 17.7

3 Macromolecular transport 17.5

4 Signal transduction 15.0

5 Nuclear pore assembly 11.7

6 Signal transduction 10.1

7 RNA processing 9.6

8 cell division 9.3

9 RAS small GTPase 7.8

10 Amino acid metabolism 7.6

11 Chemokine/cytokine 6.7

12 Iron metabolism 4.1

13 Lipid metabolism 3.5

(D): Host proteins by sonication affected by *S. aureus* during chronic phase of infection. A total 17 gene functional groups were identified by the Gene Functional Classification tool using DAVID software. The associated biology terms with each group are manually summarized based on gene-term enrichment buttons provided for each functional group.

Gene functional group no. Associated biology Group Enrichment Score

1 Cell catabolism 22.4

2 RAS small GTPase 20.9

3 Signal transduction 20.8

4 Cell transport 19.1

5 Cell respiration 16.9

6 Molecular chaperones 15.3

7 Amino acid metabolism 15.1

8 Chemokine/cytokines 14.3

9 cell hemostasis 12.2

10 Cytoskeleton signaling 11.3

11 Membrane transport 10.7

12 Cell membrane components 10.6

13 RNA processing/splicing factors 10.1

14 Intracellular protein transport 7.8

15 Cell respiration 6.8

16 Macromolecular assembly 4.7

17 Blood hemostasis 2.1
