# Supplementary material for: Proteomics of Staphylococcus aureus biofilm matrix in a rat model of orthopedic implant-associated infection
Source: PLoS One. 2017 Nov 9;12(11):e0187981. doi: 10.1371/journal.pone.0187981 (PMC5679556; doi:10.1371/journal.pone.0187981)
Supplement: S6 Table — (DOCX) [file pone.0187981.s006.docx]

Table S6. Oligonucleotide primers used in this study

Primers Sequence

ebh1 gatgatatcgtcgacGGATCAGAATTAGGTGTTACCTCAC

ebh2 TAGCGTTAATCGGTCATTTACACGAgcggccgcGTTCACTCCTTATCTTGTTGTTATGT

ebh3 ACATAACAACAAGATAAGGAGTGAACgcggccgcTCGTGTAAATGACCGATTAACGCTA

ebh4 gatgatatcgaattcATTTGGTGCAGCATTAATTTGTTGC

sasF1 gatgatatcgtcgacGGTAGTGATGTTTTGGTATGGC

sasF2 CTTATTGCGTCGTGATAACCgcggccgcATTGAAACGGTTTCCCTCGATA

sasF3 TATCGAGGGAAACCGTTTCAATgcggccgcGGTTATCACGACGCAATAAG

sasF4 gatgatatcgaattcAAGTATAAACAAGGAGTTCGGAC

erm11 cccgggAATTGAATGAGACATGCTAC

erm12 aagcttAAAACTGGTTTAAGCCGAC
